# Prevalence and Application of Speech-Language Therapy in Individuals With Klinefelter Syndrome: A Scoping Review

**DOI:** 10.7759/cureus.85523

**Published:** 2025-06-07

**Authors:** Jordanna Laing, Ziad Kadoura, Brendan Guhle

**Affiliations:** 1 Research, Klinefelter Support Society, Edmonton, CAN; 2 Psychology, University of Alberta, Edmonton, CAN; 3 Biology, University of Alberta, Edmonton, CAN

**Keywords:** communication disorders, early intervention, klinefelter syndrome, speech-language therapy, xxy

## Abstract

Klinefelter syndrome (KS), a condition resulting from the presence of an extra X chromosome in males (47,XXY), is associated with a high prevalence of communication difficulties, however, the application and evaluation of speech-language therapy (SLT) in this population remains poorly understood. Despite extensive documentation of language impairments in individuals with KS, no comprehensive synthesis of SLT interventions or outcomes has been conducted. To address this gap, we conducted a scoping review mapping the existing literature on SLT in individuals with KS to inform future clinical practice and research.

A systematic search was performed across four databases: PubMed, Embase, PsycINFO, and Scopus. Eligible studies discussed SLT in relation to individuals with KS. The search identified 57 articles, which were narrowed down to four articles that met the inclusion criteria; the findings were narratively synthesized.

Following data extraction, thematic analysis revealed three overarching themes within the literature: (1) High utilization of SLT in KS, (2) Early interdisciplinary intervention for language deficits, and (3) Access barriers and lack of outcome data.Findings highlight that SLT is one of the most frequently accessed early interventions for children with KS, yet therapeutic outcomes are rarely evaluated. Early interdisciplinary intervention models were emphasized as crucial for addressing the complex language needs associated with KS; however, barriers to timely access were prevalent, including limited provider awareness and insufficient knowledge of KS. Moreover, while high rates of SLT usage were reported, few studies assessed the effectiveness or long-term outcomes of interventions, emphasizing the need for further research in this area.

The findings of this scoping review suggest that improving awareness among healthcare providers and educators, along with ongoing evaluation of SLT outcomes, is essential to optimize developmental support for children with KS.

## Introduction and background

Effective communication is fundamental to cognitive, social, and emotional development. However, individuals with Klinefelter syndrome (KS), a genetic condition characterized by the presence of an extra X chromosome (47,XXY) that affects approximately one in 600 male births, frequently experience challenges in speech, language, and social communication [1]. Clinically, KS is associated with a range of features, including hypogonadism, infertility, and varying degrees of cognitive and behavioral challenges [1]. Studies have reported that up to 92% of children with KS exhibit communication impairments, including deficits in expressive and receptive language, phonological processing, and pragmatic language skills [2].

These language difficulties often persist into adolescence and adulthood, impacting academic achievement, social integration, and overall quality of life. Research indicates that individuals with KS may struggle with high-level language competencies and Theory of Mind, affecting their ability to comprehend metaphors and engage in complex inferential communication [3]. Furthermore, the neuropsychological profile of KS shares similarities with specific language impairment, suggesting overlapping etiological pathways [4].

Concerns regarding language development in individuals with KS are not new. As early as the 1980s, Walzer and colleagues published preliminary observations identifying expressive language delays and learning difficulties among boys with XXY [5]. Their work was among the first to empirically document language-related challenges in this population, pointing to a clear need for early intervention. 

Despite this early recognition, there is a lack of research focusing specifically on speech-language therapy (SLT) interventions tailored for individuals with KS. While early hormonal treatment has shown promise in enhancing language development in young boys with KS [6], the evidence base for SLT approaches remains fragmented. Existing studies often address broader neurodevelopmental aspects or focus on related conditions without addressing targeted SLT strategies for the KS population.​

A preliminary search of PROSPERO, the Cochrane Database of Systematic Reviews, and PubMed was conducted in April 2025. No relevant completed or ongoing scoping or systematic reviews on this topic were identified. With this in mind, we conducted a scoping review that mapped the extent and nature of research on speech-language therapy interventions for individuals with Klinefelter syndrome. Our objective was to synthesize what is known about SLT use, delivery models, and outcomes in this population, and to identify gaps that should guide future research.

## Review

Methods

Protocol and Registration

This scoping review followed Arksey and O’Malley's five-stage framework, which includes identifying the research question, identifying relevant studies, study selection, charting the data, and collating, summarizing, and reporting the results [7]. The review also incorporated the enhanced methodology for scoping reviews outlined by the Joanna Briggs Institute [8]. The protocol was developed in accordance with the Preferred Reporting Items for Systematic Review and Meta-Analysis Protocols (PRISMA-P) and PRISMA for Scoping Reviews (PRISMA-ScR) checklists and was prospectively registered with the Open Science Framework on April 29, 2025 [9]. The review was conducted in alignment with this protocol.

Framework Stage 1: Identifying the Research Question

A systematic literature search was conducted on SLT in individuals with Klinefelter syndrome. The research question was: “What is known about the use and outcomes of SLT in people with Klinefelter syndrome?”

Framework Stage 2: Identifying Relevant Studies

The research team collectively agreed upon the inclusion criteria, search terms, and databases to be used in the scoping review. A comprehensive search was conducted by three reviewers (JL, ZK, and BG) across four databases: PubMed, Embase, PsycINFO, and Scopus. Grey literature was identified through targeted searches on platforms such as Google Scholar and professional organizations (e.g., American Speech-Language-Hearing Association (ASHA), Royal College of Speech and Language Therapists (RCSLT)). These databases were searched from their inception to May 5th, 2025. The primary keywords utilized in the search strategy were: “Klinefelter Syndrome”, “XXY”, “47,XX7”, “Speech therapy”, “Language intervention”, and “Speech language pathology”. Keywords were combined in different ways by using Boolean operators of AND to combine concepts and OR to include synonyms.

Framework Stage 3: Study Selection

Eligibility criteria were established to ensure the inclusion of studies that specifically addressed the use and outcomes of speech-language therapy or communication-focused interventions in individuals with Klinefelter syndrome. Studies were eligible for inclusion if they involved individuals of any age diagnosed with KS and examined speech-language therapy or other language or communication-based interventions. To capture a broad range of relevant information, we included empirical studies, clinical guidelines, and grey literature published in English. Studies were excluded if they focused on populations without KS, did not relate to speech-language therapy, were non-empirical (e.g., opinion pieces), or were published in languages other than English. 

Search results were imported into Covidence, a web-based application that streamlines reviews, where duplicates were removed using the built-in duplicate detection function. A two-step process was implemented; first, two independent reviewers (JL and ZK) screened the titles and abstracts of all articles, labelling each as “Yes”, “Maybe” or “No”. Articles marked as “Yes” by both reviewers were considered for full-text review, whereas articles marked as “Yes” by one reviewer or “Maybe” by both reviewers were discussed to reach a consensus. An independent third reviewer (BG) resolved any persisting disagreements. In the second stage, JL and ZK conducted full-text reviews of all included articles, with BG again resolving any conflicts.

Framework Stage 4: Charting the Data

Data from the included articles were extracted by JL using a standard data extraction form, which captured demographic data (author(s), year, study location, study type), population studied, and key findings relating to prevalence of speech/language therapy use, accessibility (e.g., barriers, availability), caregiver perspectives, and observed outcomes or changes in language development.

Framework Stage 5: Collating, Summarizing and Reporting Results

Based on the charted data, a narrative review was generated, including a table summarizing study characteristics (Table 1) and a thematic analysis of key findings. This involved qualitatively reviewing each article’s key findings related to prevalence of speech/language therapy use, accessibility (e.g., barriers, availability), caregiver perspectives, and observed outcomes or changes in language development, which were then categorized into overarching themes.

**Table 1:** Overview of studies included SCA: sex chromosome aneuploidy, SLT: speech-language therapy

Author(s)	Year	Country	Study Design	Population Studied	Key Findings
Bishop et al. [10]	2011	United Kingdom	Cross-sectional observational	Parents/caregivers of children aged 4-16 years with a prenatal diagnosis of sex chromosome trisomy; 19 47,XXY boys included	47% had accessed SLT. Pragmatic language difficulties and reduced conversational reciprocity were observed, suggesting SLT need even in the absence of formal autism diagnoses.
Tartaglia et al. [11]	2015	United States	Descriptive cross-sectional survey	74 boys with 47,XXY, part of a broader SCA cohort including children and adolescents (reported ages ranged from 8 months to 14 years in case examples).	37.8% received additional therapies (e.g., SLT) following multidisciplinary evaluation. Participation in specialized clinics (e.g., eXtraordinarY Kids Clinic) facilitated access to services beyond school.
Graham et al. [12]	1988	United States	Prospective longitudinal cohort	14 boys with 47,XXY, aged 5-12 yrs	All received school-based SLT. Persistent deficits in language production and auditory processing. SLT strategies recommended included visual supports, simplified instructions, and structured vocabulary programs. Articulation screenings often passed through conscious effort.
Thompson et al. [13]	2020	United States	Cross-sectional mixed-methods survey	Parents/caregivers of children with a confirmed SCA diagnosis who had not yet entered Kindergarten, nearly evenly split between infant/toddler (n=52) and preschool (n=53) age groups. Of these, 72 were caregivers of children with a postnatally confirmed XXY diagnosis.	58.3% of boys with XXY received public early intervention (including SLT). SLT was the most common service among infants (43.8%) and preschoolers (45.3%). Barriers included limited provider knowledge and failure to qualify based on borderline scores. Parents viewed SLT as beneficial when accessible.

Results

**Figure 1:**
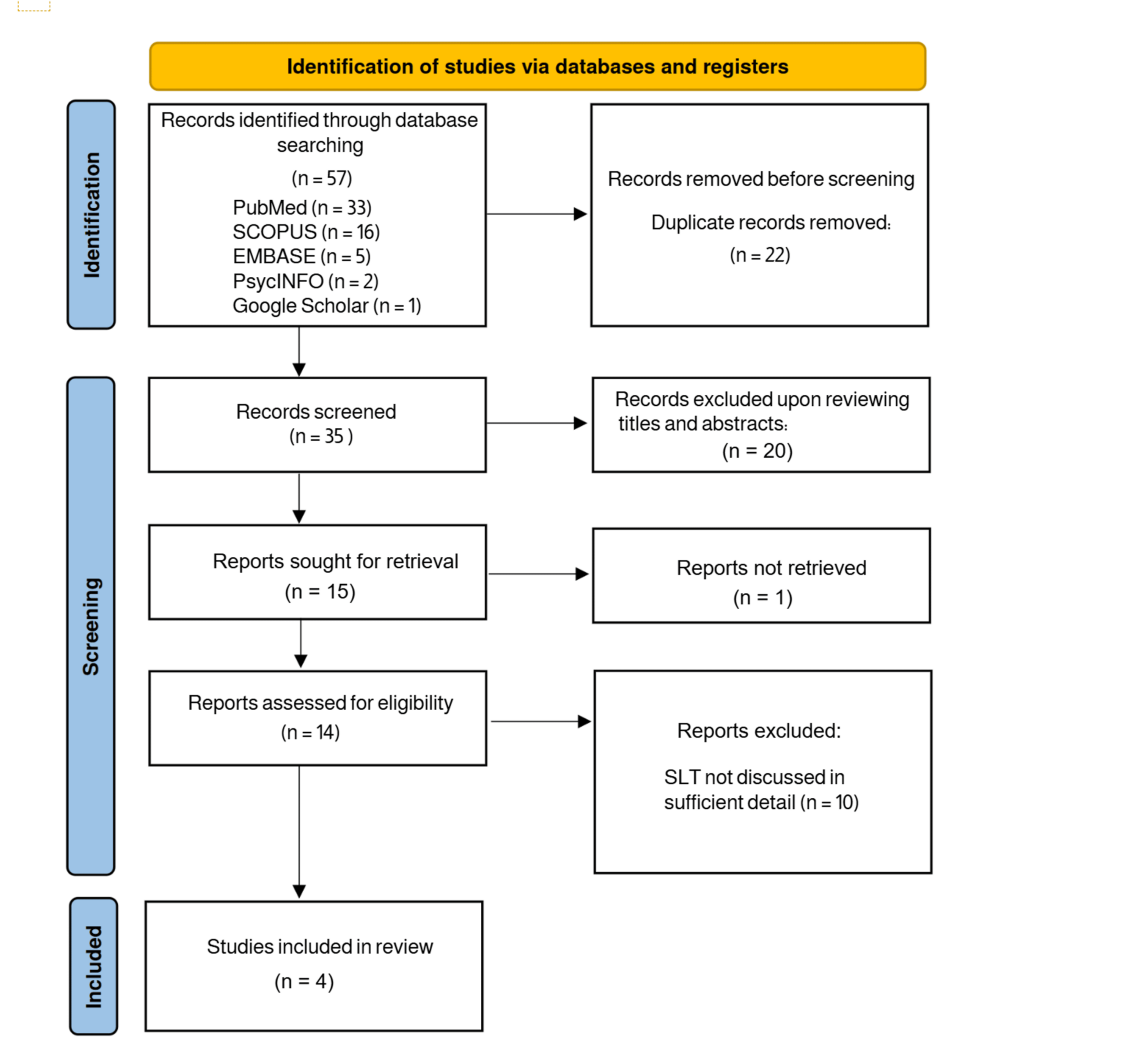
PRISMA diagram of scoping review identification, screening, and inclusion. PRISMA: Preferred Reporting Items for Systematic Reviews and Meta-Analyses, SLT: speech-language therapy

A total of 57 articles were identified through the preliminary database and grey literature searches. Following the removal of duplicate studies (n=22), the first screening phase led to the exclusion of 20 articles, leaving 15 articles for full-text screening. Ultimately, four studies met the inclusion criteria and were included in the final review, as reported in Figure 1.

The included studies were published between 1988 and 2020 and originated from the United States and the United Kingdom. Study designs included cross-sectional surveys [11,13], a longitudinal cohort study [12], and an observational report [10]. All studies included individuals with KS though some encompassed broader sex chromosome aneuploidy (SCA) populations.

Thematic Findings

Three overarching themes were identified.

High utilization of SLT in KS: A prominent theme across the literature was the high prevalence of SLT use among children with KS, particularly in early childhood. SLT was one of the most frequently accessed early interventions for children with KS. Between 43.8% and 58.3% of children with 47,XXY received early SLT services [13]. Bishop et al. reported that nearly half (47%) of boys with KS had accessed SLT [10]. Despite the high rates of use, therapeutic outcomes were rarely evaluated. Graham et al. briefly noted that boys receiving SLT were sometimes able to pass articulation screenings through focused effort, but no long-term outcome data were provided [12].

Early interdisciplinary intervention for language deficits: Several studies emphasized the importance of early, multidisciplinary intervention. Tartaglia et al. highlighted the eXtraordinarY Kids Clinic, which integrates SLT with pediatric and neurodevelopmental services [11]. Graham et al. and Thompson et al. both pointed to the need for early screening and individualized care, noting that children with KS often have subtle language deficits not detected through standard assessments [12,13]. Bishop et al. reported pragmatic and expressive language difficulties even in children without other diagnoses, reinforcing the need for specialized evaluation protocols [10].

Access barriers and lack of outcome data: Access to SLT was frequently hindered by limited provider awareness and inconsistent referral practices. Thompson et al. found that few early intervention providers were knowledgeable about SCTs, and children with borderline test scores often failed to qualify for therapy [13]. Parents expressed frustration with having to wait for academic failure before accessing services, despite a confirmed KS diagnosis. Only 11 of 50 U.S. states list 47,XXY as an automatically qualifying condition for early intervention [13]. While some programs, such as the eXtraordinarY Clinic, helped families secure SLT services [11], few studies examined the impact of these interventions. Graham et al. was the only study to recommend specific SLT techniques [12]. The overall lack of outcome-focused research represents a significant gap in evidence-based practice.

Discussion

Recent research on KS has increasingly highlighted the significance of early, targeted interventions to address the developmental challenges associated with this condition. As a sex chromosome aneuploidy, 47,XXY impacts various aspects of physical and cognitive development, including language, motor skills, and social integration. However, many of these challenges remain subtle in early childhood, making timely identification and intervention a crucial factor in optimizing long-term outcomes. 

This scoping review provides the first comprehensive synthesis of literature on the use and outcomes of speech-language therapy in individuals with Klinefelter syndrome. The findings reveal that although SLT is widely accessed, particularly in early childhood [10,13], there is a notable lack of research evaluating its effectiveness or defining optimal intervention strategies [12]. 

The consistent identification of SLT as a core intervention suggests a strong recognition of communication challenges in KS. However, the lack of longitudinal and outcome-focused studies indicates a missed opportunity to build an evidence base for what works. Although some studies noted where SLT was delivered - for example, Graham et al. reported exclusively school-based therapy [12], and Tartaglia et al. described a specialized multidisciplinary clinic [11] - none disaggregated outcomes by service setting. As a result, we cannot infer whether school-based versus clinic-based SLT yields differential gains in language skills, therapy intensity, or caregiver satisfaction in children with SCA. Future research should explicitly compare these delivery models to inform best-practice recommendations. Graham et al. was the only study to offer concrete strategies such as visual aids and narrative-focused interventions, yet this work remains dated and has not been followed by large-scale replication [12]. 

Moreover, the value of interdisciplinary care is repeatedly emphasized. Integrated clinics, like the one described by Tartaglia et al., demonstrate the potential of team-based approaches to capture nuanced developmental needs [11]. Yet, access to such models is not widespread, and delays in diagnosis or referral can limit early intervention opportunities. Subtle deficits, such as difficulties with inferential language or conversational reciprocity, often go unnoticed by general practitioners and educators, further delaying support [10].

Systemic access barriers remain a critical issue. Thompson et al. found that few U.S. states automatically qualify 47,XXY as an eligible diagnosis for early intervention, and many children must show academic failure before support is approved [13]. This reactive model conflicts with the developmental nature of communication disorders and runs counter to best practices in early childhood intervention.

In conclusion, the findings underscore the need for targeted, syndrome-specific research on SLT in KS. Future studies should assess intervention effectiveness, identify optimal therapy components, and examine access disparities. Expanding provider education and embedding SLT within comprehensive developmental frameworks may enhance early identification and outcomes for individuals with Klinefelter syndrome.

Limitations

A primary limitation of this scoping review is the inclusion of studies that examined heterogeneous SCAs, such as 47,XXX and 47,XYY, rather than focusing exclusively on Klinefelter syndrome. This grouping limits the ability to isolate SLT usage patterns and access barriers specific to KS. As a result, prevalence data may reflect broader SCA trends, potentially obscuring condition-specific insights. Future research should prioritize well-defined KS cohorts to generate more targeted evidence.

A second limitation concerns the age range of study populations. Most studies focused on early childhood or early adolescence, with little attention to older adolescents or adults. Since communication challenges in KS can persist or change across the lifespan, this limits the generalizability of findings. Broader age representation is needed to evaluate the long-term efficacy and relevance of SLT for individuals with KS, particularly in adulthood.

Finally, this review included only English-language studies, primarily from the U.S. and U.K., because only these met our stringent inclusion criteria. Studies from other regions were excluded due to insufficient detail on SLT interventions, language barriers, or other methodological issues. As a result, our findings may not fully reflect the cultural and systemic diversity of non-English-speaking or non-Western populations. In addition, none of the included studies provided information on the ethnic backgrounds of participants, which limits the generalizability of the findings across diverse populations.

Future research should focus on KS-specific populations, include broader age ranges across the lifespan, and incorporate studies from diverse linguistic and cultural contexts to enhance generalizability and relevance.

## Conclusions

This scoping review provides the first synthesized overview of existing research on the use and outcomes of SLT in individuals with Klinefelter syndrome (47,XXY). The findings demonstrate that while SLT is widely utilized, particularly in early childhood, the evidence base supporting its effectiveness remains limited and fragmented. Early, interdisciplinary interventions appear to hold promise in addressing the complex and often subtle language difficulties associated with KS, but access remains inconsistent due to limited provider awareness, diagnostic delays, and systemic barriers. Moreover, despite the high prevalence of SLT use, few studies evaluate therapeutic outcomes or define best practices tailored to this population.

The review underscores a pressing need for more targeted research focusing exclusively on Klinefelter syndrome, with careful attention to age diversity and outcome measurement. Future studies should adopt longitudinal and intervention-based designs to assess the long-term efficacy of SLT and to identify evidence-based strategies suited to the unique language profiles of individuals with KS. Additionally, efforts to improve provider education and increase diagnostic recognition are essential to ensuring timely and equitable access to language support services. By addressing these gaps, future research can inform more effective, individualized care and ultimately enhance the communication outcomes and quality of life for individuals with Klinefelter syndrome.
